# Machine Learning Prediction of Lymph Node Metastasis in Breast Cancer: Performance of a Multi-institutional MRI-based 4D Convolutional Neural Network

**DOI:** 10.1148/rycan.230107

**Published:** 2024-04-12

**Authors:** Dogan S. Polat, Son Nguyen, Paniz Karbasi, Keith Hulsey, Murat Can Cobanoglu, Liqiang Wang, Albert Montillo, Basak E. Dogan

**Affiliations:** From the Department of Diagnostic Radiology (D.S.P., K.H., A.M., B.E.D.), Lyda Hill Department of Bioinformatics (S.N., P.K., M.C.C., L.W., A.M.), and Biomedical Engineering Department (A.M.), University of Texas Southwestern Medical Center, 5323 Harry Hines Blvd, Dallas, TX 75390-8585.

**Keywords:** MR Imaging, Breast, Breast Cancer, Breast MRI, Machine Learning, Metastasis, Prognostic Prediction

## Abstract

**Purpose:**

To develop a custom deep convolutional neural network (CNN) for noninvasive prediction of breast cancer nodal metastasis.

**Materials and Methods:**

This retrospective study included patients with newly diagnosed primary invasive breast cancer with known pathologic (pN) and clinical nodal (cN) status who underwent dynamic contrast-enhanced (DCE) breast MRI at the authors’ institution between July 2013 and July 2016. Clinicopathologic data (age, estrogen receptor and human epidermal growth factor 2 status, Ki-67 index, and tumor grade) and cN and pN status were collected. A four-dimensional (4D) CNN model integrating temporal information from dynamic image sets was developed. The convolutional layers learned prognostic image features, which were combined with clinicopathologic measures to predict cN0 versus cN+ and pN0 versus pN+ disease. Performance was assessed with the area under the receiver operating characteristic curve (AUC), with fivefold nested cross-validation.

**Results:**

Data from 350 female patients (mean age, 51.7 years ± 11.9 [SD]) were analyzed. AUC, sensitivity, and specificity values of the 4D hybrid model were 0.87 (95% CI: 0.83, 0.91), 89% (95% CI: 79%, 93%), and 76% (95% CI: 68%, 88%) for differentiating pN0 versus pN+ and 0.79 (95% CI: 0.76, 0.82), 80% (95% CI: 77%, 84%), and 62% (95% CI: 58%, 67%), respectively, for differentiating cN0 versus cN+.

**Conclusion:**

The proposed deep learning model using tumor DCE MR images demonstrated high sensitivity in identifying breast cancer lymph node metastasis and shows promise for potential use as a clinical decision support tool.

**Keywords:** MR Imaging, Breast, Breast Cancer, Breast MRI, Machine Learning, Metastasis, Prognostic Prediction

*Supplemental material is available for this article.*

Published under a CC BY 4.0 license.

## Graphical abstract

**Figure:**
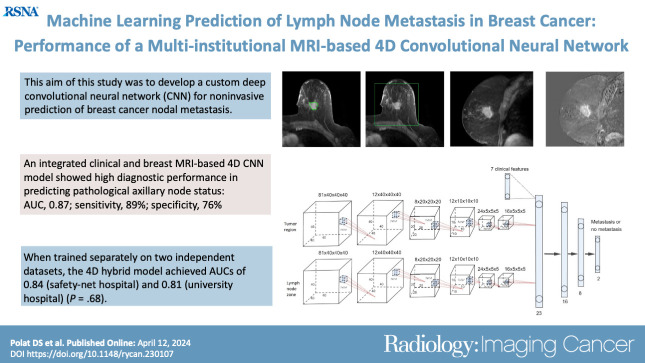

**Summary**

A highly sensitive MRI-based four-dimensional convolutional neural network model showed high performance in predicting axillary lymph node metastasis in patients with breast cancer and may help reduce the complications of unnecessary interventional and surgical procedures.

**Key Points**

■ An integrated clinical and breast MRI–based four-dimensional (4D) convolutional neural network model for predicting pathologic axillary node status showed high diagnostic performance, with area under the receiver operating characteristic curve (AUC), sensitivity, and specificity values of 0.87, 89%, and 76%, respectively.
■ A machine learning model using clinicopathologic features alone demonstrated lower predictive performance of pathologic node status (AUC, sensitivity, specificity, and false-negative rate values of 0.63, 75%, 52%, and 65%, respectively)
■ The 4D hybrid model showed similar diagnostic performance (*P* = .68) on two independent data sets from institutions with variable patient and cancer profiles; when trained on data from the safety-net hospital, the model achieved an AUC of 0.84 (95% CI: 0.75, 0.91), while when trained on university hospital data, the model achieved an AUC of 0.81 (95% CI: 0.73, 0.87).

## Introduction

Breast cancer is the leading cause of cancer death in female individuals, responsible for approximately 40 000 deaths annually in the United States. Most deaths are due to metastatic disease, for which the first site is usually an ipsilateral axillary lymph node (1,2). Nodal status is one of the most important factors determining disease prognosis and guiding treatment decisions such as neoadjuvant chemotherapy, radiation therapy, and axillary lymph node dissection (3,4). There are two strategies to identify axillary lymph node metastasis: *(a)* a combination of clinical examination, imaging, and imaging-guided biopsy, which determines clinical node (cN) stage; or *(b)* surgical staging with sentinel lymph node biopsy (SLNB) or axillary dissection to determine the pathologic node (pN) stage (3). Preoperative US with guided needle biopsy helps identify a median of 50% of patients with axillary nodal metastases prior to SLNB (5,6). Variations in scan technique and criteria for biopsy and the inability to detect and sample small metastasis limits the reproducibility of US (7–12). Selected patients undergo breast MRI, which allows axillary assessment but has a mean sensitivity of only 60% (range, 33.3%–97%) and negative predictive value of 80% (range, 1.9%–99.5%) (13) in detecting pN stage. Hence, patients with benign findings from axillary imaging or biopsy routinely undergo surgical SLNB. SLNB has higher sensitivity compared with imaging, ranging from 86% to 92% (14), but it is a diagnostic procedure that involves morbidity associated with anesthesia, surgery, and radiation exposure because of radiopharmaceuticals used to identify the sentinel node (4,15,16). Permanent adverse effects of SLNB are reported as subclinical lymphedema in 24.4% of patients (17), clinical lymphedema (mean, 5.6% [range, 0%–11%]) (18), chronic axillary pain in 16%, sensory disorders including paresthesia (2%–22%), and limited arm motion (0%–9%) (19). Clinical predictions of node-positive status are primarily focused on nonsentinel node positivity and do not allow omission of SLNB (20,21). However, these methods do not address the overtreatment of a large proportion of patients (52%–82%) who undergo surgical diagnosis of the axilla (22,23). Therefore, a robust imaging-based predictive model that assesses the presence of lymph node metastasis with high sensitivity would help select patients who could potentially avoid SLNB and facilitate treatment decisions for patients with breast cancer, without the need for an invasive procedure. Identifying cN stage is important as cN+ status can be the determining factor in whether the patient will be treated with neoadjuvant therapy versus upfront surgery. Studies this decade in patients with early-stage breast cancer suggest that deep learning models trained with either US or MR images can yield acceptable diagnostic performance in predicting ipsilateral nodal metastasis (24,25). However, it is unclear whether these models developed based on lower-stage category T1 or T2 breast cancers would be applicable to all breast cancers. Furthermore, these models are trained with single-institutional and single–MRI unit data sets, precluding their uniform application (26–29). These models also suffer from assumptions inherent to black box optimization hyperparameter tuning tasks, which preclude their robust application to imaging data sets from other institutions.

In this study, we aimed to develop a deep learning algorithm to predict cN and pN stages regardless of tumor size by using breast MR image data sets from two different MRI units and institutions with racially diverse population data sets. Heteroscedastic evolutionary Bayesian optimization (HEBO) is advantageous over traditional Bayesian optimization and hyperband in hyperparameter optimization because of its greater adaptability to complex and varied problems, akin to how a radiologist tackles diverse cases. It efficiently explores hyperparameter spaces, making it quicker to find the optimal settings, much like efficiently diagnosing with limited images. Additionally, HEBO excels with limited data and handles uncertainty well, offering more personalized and flexible optimization, which is essential in real-world applications where data can be noisy or incomplete. This makes HEBO a more versatile and reliable choice in various scenarios, much like a radiologist’s tailored approach to each patient. We aimed to determine whether our predictive model based on dynamic tumor MR images and selected clinicopathologic variables, which also integrates patch-based learning and hyperparameter optimization techniques (HEBO) that offset small-volume data, would yield high diagnostic performance in noninvasively diagnosing axillary metastasis, exceeding that of the currently available diagnostic imaging technologies used in clinical standard of care.

## Materials and Methods

### Patient Cohort

**Figure 1:**
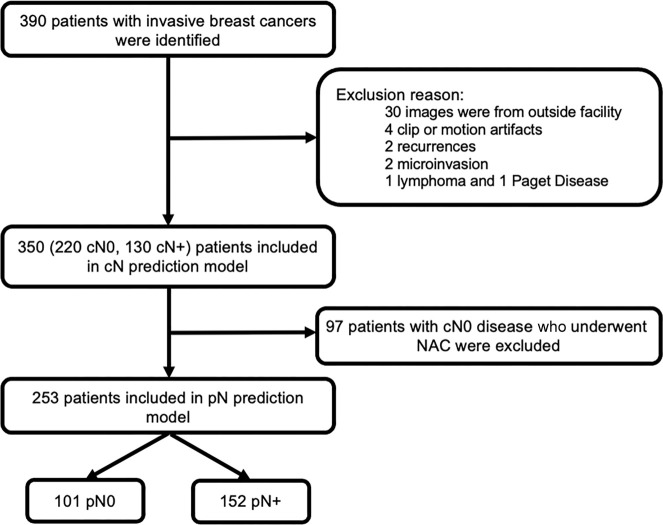
Consort flow diagram. cN = clinical nodal stage, NAC = neoadjuvant chemotherapy, pN = pathologic nodal stage.

Written informed consent was waived for this institutional review board–approved, Health Insurance Portability and Accountability Act–compliant retrospective analysis. Consecutive patients with primary invasive breast cancer who underwent dynamic contrast-enhanced (DCE) breast MRI as part of their initial extent of disease evaluation between July 2013 and June 2016, performed in a university hospital and a safety-net hospital within the same health care network, were retrospectively reviewed. Inclusion and exclusion criteria for patients are summarized in Figure 1. Recurrent cancers and inflammatory (cT4b) breast cancers replacing entire breast tissue were excluded. Age, race, tumor size, clinical regional nodal metastasis (cN1–3), tumor stage, and neoadjuvant chemotherapy history were collected from electronic health records. Grade (Nottingham histologic), estrogen receptor (ER) and human epidermal growth factor receptor 2 (HER2) status (positive vs negative), and Ki-67 index were obtained from standardized College of American Pathologists pathology reports using previously published criteria (Fig S1).

### DCE MRI Technique and Image Segmentation

All breast MRI examinations were performed with patients lying prone in 1.5-T scanners (Optima MR450w, GE HealthCare; or Intera, Philips Healthcare) using a dedicated eight-channel (Invivo Sentinelle; Siemens) or seven-channel (dSTream; Philips Healthcare) coil. A single precontrast and four serial bilateral axial dynamic image sets were obtained before and immediately after rapid intravenous bolus infusion of 0.1 mmol of gadopentetate dimeglumine contrast medium (Magnevist; Bayer Healthcare Pharmaceuticals) per kilogram of body weight at a rate of 3 mL/sec with a power injector (Spectris Solaris MR Injector; Medrad), with an average dynamic temporal resolution of 90 sec/phase. The primary tumor was delineated using Horos with an OsiriX plugin (Pixmeo) using images from the second postcontrast acquisition by a fellowship-trained breast radiologist (B.E.D.) with 15 years of experience in interpreting breast MRI studies. For multifocal or multicentric disease, the index lesion used to clinically stage the patient was delineated. As this exquisite approach to finely delineate tumors requires time and expertise, we derived crude bounding boxes using existing segmentation. Our model uses those bounding boxes, which can be drawn in less than a minute.

### Staging the Axilla

The cN stage was determined using clinical and imaging assessment and imaging-guided core-needle biopsy in accordance with the American Joint Committee on Cancer staging system (30). The cN information from oncology treatment planning notes was collected. Patients staged as cN negative based on either clinical examination or a combination of MRI, axillary US, and US-guided needle biopsy findings were labeled as cN0, while patients with positive diagnosis confirmed with clinical examination and imaging-guided needle biopsy (cN1–3) were labeled cN+. The pN status was determined by first excluding all patients with cN0 status who underwent neoadjuvant chemotherapy prior to surgical axillary staging. Patients who had preoperative biopsy-verified axillary metastasis, as well as patients with cN0 status whose metastases were identified at SLNB, were labeled pN+. Patients with benign SLNB findings comprised the pN0 group.

### Data Preprocessing, Model Construction, and Interinstitutional Model Performance Comparison Tests

**Figure 2:**
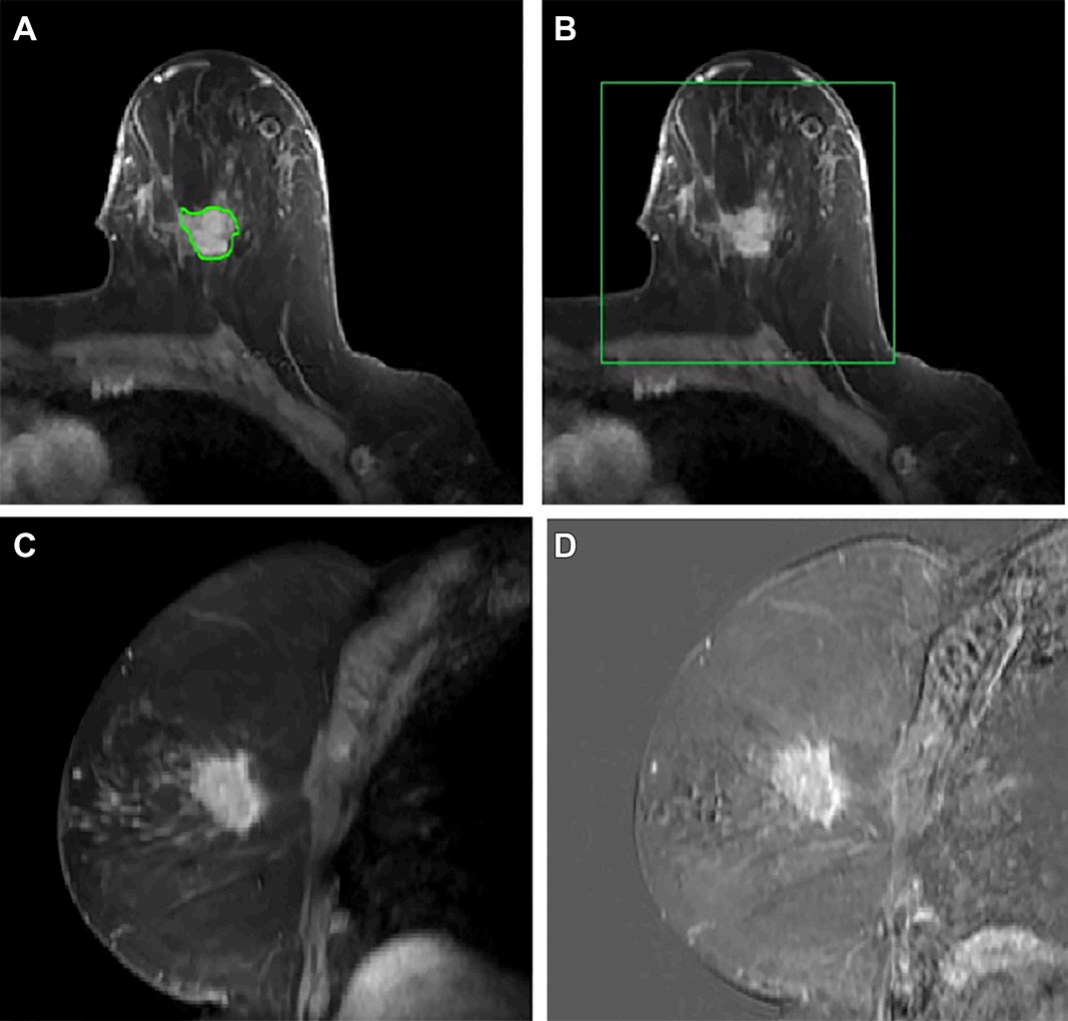
Preprocessing a volumetric dynamic contrast-enhanced MR image. **(A)** The primary tumor is radiologist delineated at time 3 in each section (green contour), though the proposed approach requires only a box around the tumor. **(B)** The MR image is cropped to a cuboidal volume around the tumor. **(C)** Sagittal view shows breast at time 1. **(D)** The tumor is enhanced by computing difference images, shown here: time 3–time 1.

All images were resampled to consistent 1-mm^3^ isotropic resolution and harmonized using histogram equalization. A cuboidal box centered on the delineated tumor and encompassing peritumoral volume was defined to crop each image to consistently sized volumes. We used a cuboidal box of 120 × 120 × 120 voxels. The model utilized both tumor and axillary pixels. For the axillary pixels, we used the same cuboidal bounding box on the ipsilateral axilla to derive pixel information. Figure 2 provides a comprehensive overview of the data preprocessing steps. Please see Appendix S1 for further details on image intensity harmonization and voxel patching, which were used to facilitate deriving the pixels that maximally contributed to the model.

**Figure 3:**
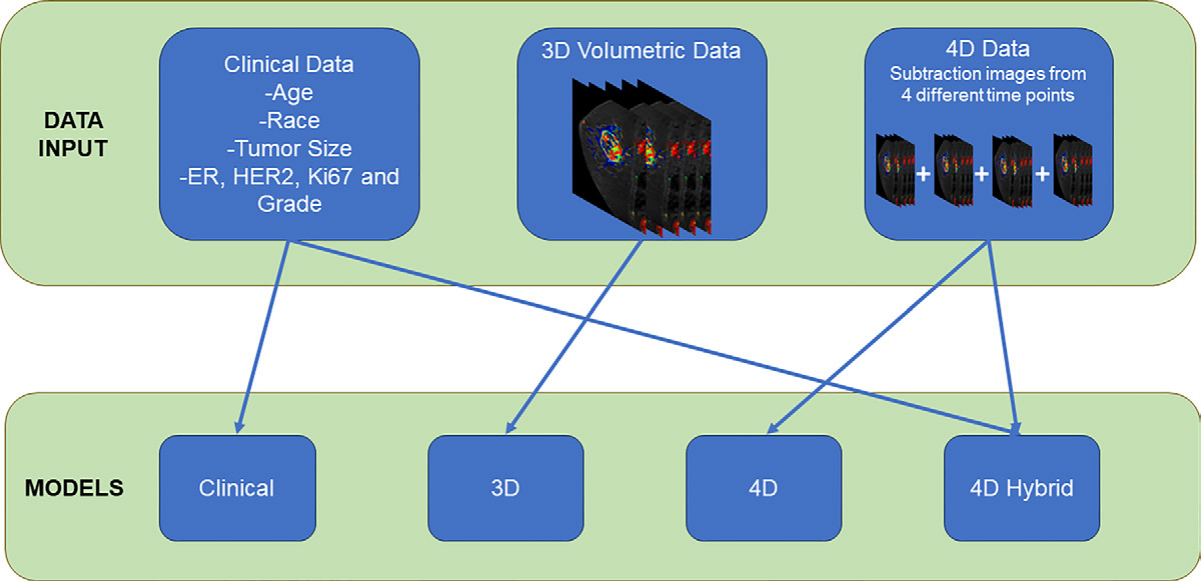
Flowchart depicts data input into the construction of the clinical, 3D volumetric imaging–, and 4D imaging–based models, as well as the hybrid 4D + clinical model. Of note, data used for model development were not used in the test data set. ER = estrogen receptor, 4D = four-dimensional, HER2 = human epidermal growth factor receptor 2, 3D = three-dimensional.

We hypothesized that the primary tumor and axilla image pixels would contain information associated with axilla metastasis status. To test this, we developed multiple convolutional neural network (CNN)–based models (31) that learn to recognize patterns in images. In the DCE MRI sequence, various temporal phases are designated as time 0 through time 5. Time 0 is identified as the phase prior to contrast enhancement, while time 1 to time 5 represent successive contrast-enhanced phases following the initial noncontrast sequence. Ablation experiments were conducted by testing a model using only clinical features, a three-dimensional (3D) model using only time 2 to time 0 volumes, an image-only model that utilizes all DCE time points (four-dimensional [4D] model), and last, a 4D hybrid model integrating difference volumes and clinicopathologic data. Diagnostic performance outcomes of each model were compared. Figure 3 presents a detailed flowchart illustrating the integration of data inputs across various models. Please see Appendix S1 for further model development and construction details.

Separate two-category classifiers were trained to predict cN0 versus cN+ and pN0 versus pN+ targets, and patient data were partitioned using nested fivefold cross-validation. Therefore, all data were partitioned into five groups, each including about 70 patients. Each fold had three training groups, one validation group, and one testing group, and one iteration was performed while rotating the test data set. Reported results are the average of the five folds. Please see Appendix S1 for further information on CNN model development.

Model performance was measured on the test data, not used for training or model selection. To gain further insight into the most important voxels, gradient class activation mapping (32) was used to generate voxel saliency maps.

To test the generalizability of our model, the 4D hybrid model for pN prediction was also trained from scratch using data from one institution at a time—university hospital versus safety-net hospital—and the model trained on one institution’s data was tested on the other institution’s for diagnostic performance.

### Statistical Analysis

Statistical analysis was performed using IBM SPSS Statistics for Windows, version 27. As an initial analysis, ER status, HER2 status, tumor grade, tumor stage, and race were compared between cN0 versus cN+ as well as pN0 versus pN+ groups using Fisher exact test. Age was compared between groups using independent samples *t* tests and Ki-67 index using the Mann-Whitney *U* test. Normality for these variables was evaluated using Shapiro-Wilk test, Q-Q plot, and skewness-kurtosis, and a *P* value less than .05 was the significance criterion.

Model performance was measured via the area under the receiver operating characteristic curve (AUC), sensitivity, and specificity. AUCs were compared using DeLong test (33). When selecting the sensitivity and specificity cutoff points, we prioritized sensitivity.

## Results

### Patient Characteristics

Of 390 patients with invasive breast cancer who underwent breast MRI, 350 female patients (mean age, 51.7 years ± 11.9 [SD]; range: 21–90 years) (Fig 1) with intact newly diagnosed breast cancer met our criteria for cN analysis. Nodal and cancer stage and clinicopathologic features are summarized in Table 1.

**Table 1:**
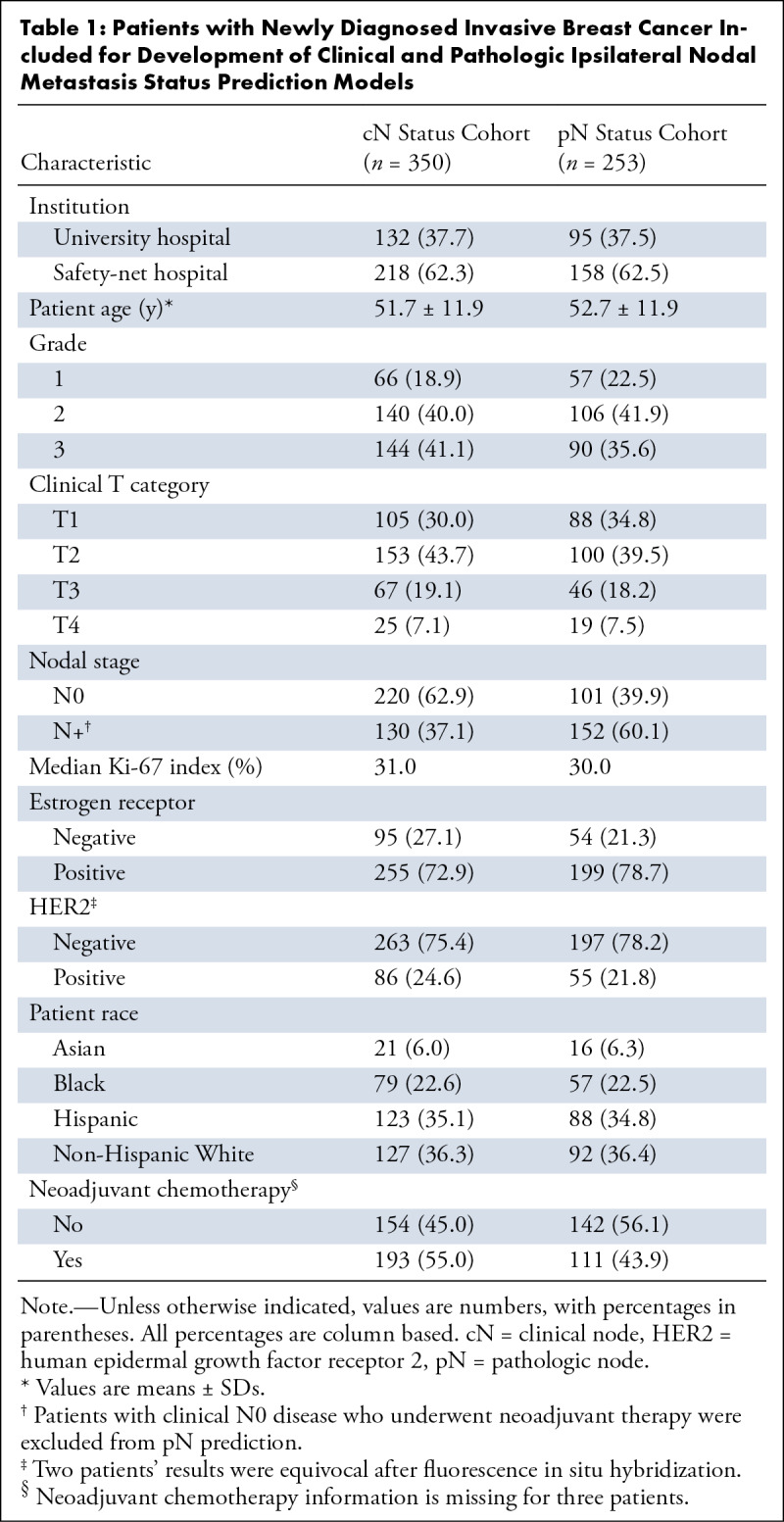
Patients with Newly Diagnosed Invasive Breast Cancer Included for Development of Clinical and Pathologic Ipsilateral Nodal Metastasis Status Prediction Models

Of 350 patients, 97 (27.7%) who had cN0 disease, as determined with imaging and needle biopsy, who underwent preoperative neoadjuvant chemotherapy were excluded from pN prediction and sensitivity analysis. Of the remaining 253 patients, 152 had pathologic evidence of metastasis, of which 118 were diagnosed with imaging plus imaging-guided needle biopsy, yielding a true-positive rate of 77.6%. In the remaining 34 patients with negative imaging or imaging-guided needle biopsy findings, SLNB showed evidence of metastasis, yielding a false-negative rate of 22.4% (34 of 152).

### Association of Clinicopathologic Factors with cN and pN Status

Correlation of clinicopathologic factors showed a significant correlation of T category with increasing prevalence of cN+ and pN+ status. Imaging sensitivity in detecting metastasis significantly increased with increasing tumor size (Table 2). The increase in sensitivity was especially significant between T1 and T2 cancers (27.8% vs 81.7%). T2 cancers were associated with significantly higher odds of cN+ disease (92% vs 7.5%, odds ratio: 13.6 [95% CI: 5.2, 35.4]; *P* < .001] and false-negative imaging findings (72.2% vs 18.3%) compared with T1 cancers (Table S3). Patients with cN+ disease were significantly younger (mean age, 40.0 vs 52.7 years; *P* = .04) than patients with cN0 disease. While the cN+ group had a higher rate of grade 3 (*P* < .001) and HER2-positive tumors (55.8% vs 44.2%, odds ratio: 2.8 [95% CI: 1.7, 4.6]; *P* < .001), they also had higher mean Ki-67 index (46.9 vs 37.4, *P* < .001). There was no evidence of a significant association of race and ER positivity with cN status (Table 3).

**Table 2:**
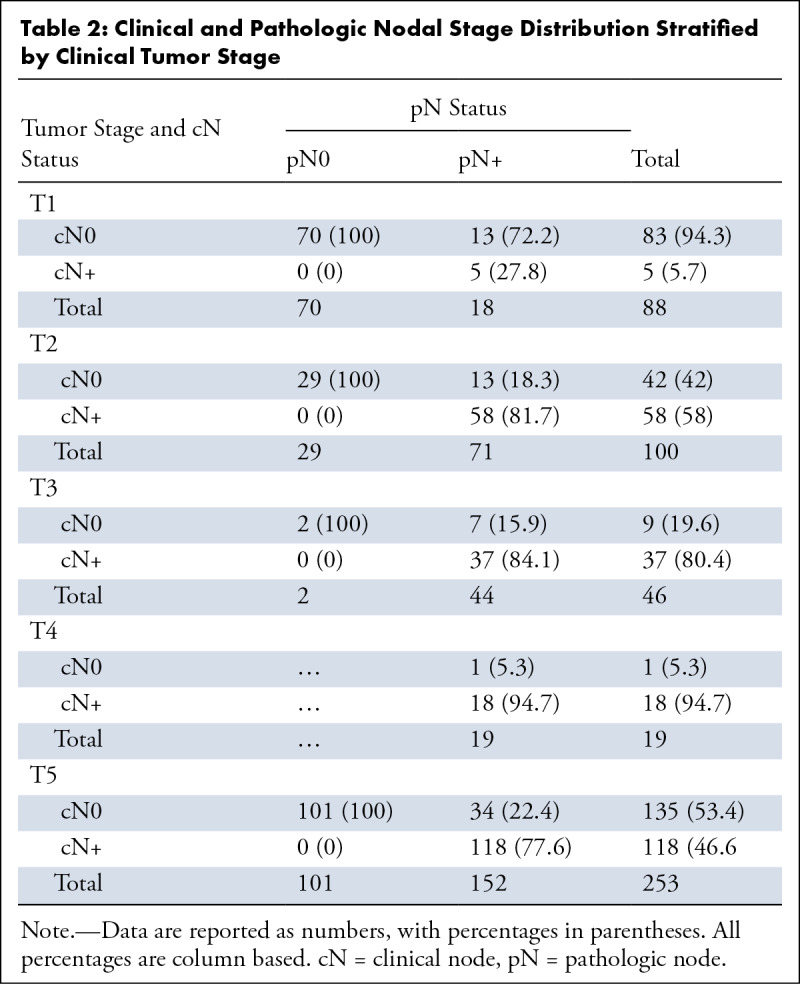
Clinical and Pathologic Nodal Stage Distribution Stratified by Clinical Tumor Stage

**Table 3:**
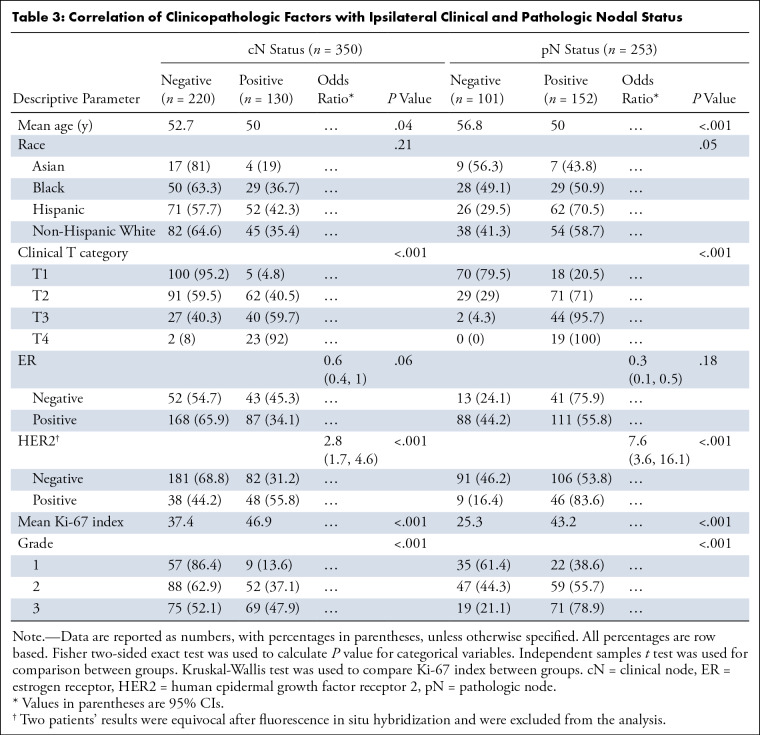
Correlation of Clinicopathologic Factors with Ipsilateral Clinical and Pathologic Nodal Status

We found similar associations with pN status. While patients with pN+ disease were younger (mean age, 50.0 vs 56.8 years; *P* < .001) than patients with pN0 disease, they also showed higher rates of T2–T4 tumors (*P* < .001). When only T1 and T2 tumors were compared, pN+ had higher rates of T2 cancers. HER2 positivity (83.6% vs 16.4%, odds ratio: 7.6 [95% CI: 3.6, 16.1]; *P* < .001) and mean Ki-67 index (43.2 vs 25.3, *P* < .001) were also higher in the pN+ group (Table 3). pN positivity correlated with increasing tumor grade (38.6% for grade 1, 55.7% for grade 2, and 78.9% for grade 3; *P* < .001). There was no evidence of a significant association between race and ER positivity with pN status.

### Ablation Tests

Our machine learning model using clinicopathologic measures alone (ie, using age, race, ER status, HER2 status, Ki-67 index, and tumor grade) predicted cN status with an AUC, sensitivity, specificity, and false-negative rate of 0.55, 35%, 77%, and 65% and predicted pN status with values of 0.63, 75%, 52%, and 25%, respectively.

The imaging-based 3D model had AUC, sensitivity, specificity, and false-negative rate values of 0.67, 67%, 66%, and 33% for cN and 0.69, 77%, 53%, and 23% for pN, respectively. The 4D model that incorporated temporal data had values of 0.70, 81%, 41%, and 19% for cN and 0.71, 77%, 51%, and 23% for pN, respectively.

### Performance of the Proposed Imaging Plus Clinicopathologic Hybrid 4D CNN Model

For the prediction of cN0 versus cN+ status, the 4D hybrid model yielded higher performance than the 3D model along the primary AUC performance measure with its AUC of 0.79 (95% CI: 0.76, 0.82) (*P* = .004) (Table 4). Additionally, the model yielded a high sensitivity of 80% (95% CI: 77%, 84%), specificity of 62% (95% CI: 58%, 67%), and a low false-negative rate of 20% (95% CI: 16%, 23%) (Table 4).

**Table 4:**
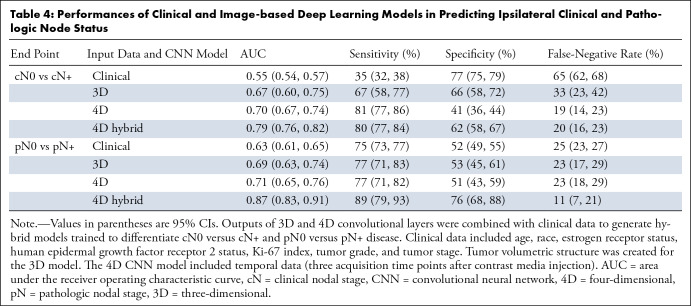
Performances of Clinical and Image-based Deep Learning Models in Predicting Ipsilateral Clinical and Pathologic Node Status

**Figure 4:**
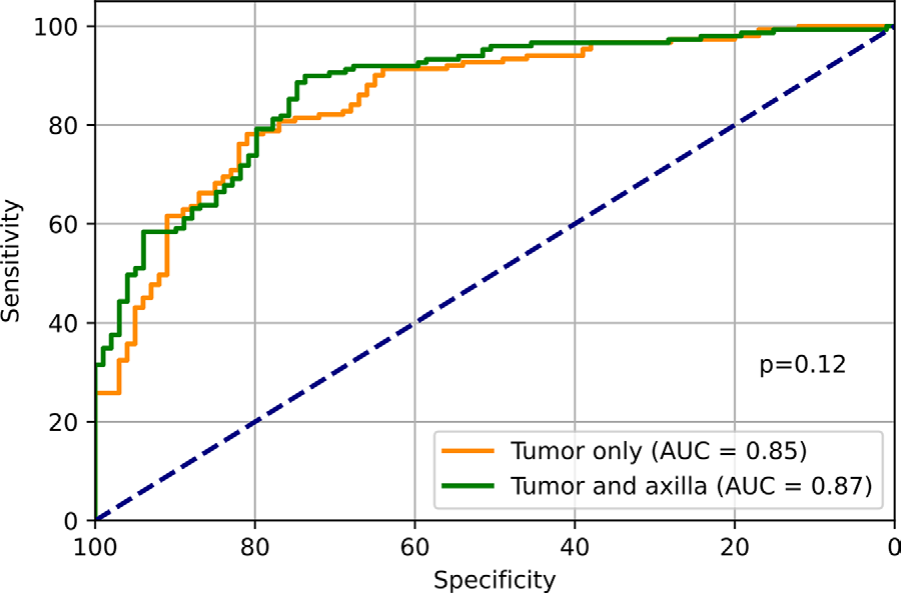
Diagnostic performance of four-dimensional hybrid model to predict ipsilateral metastatic versus benign clinical and pathologic lymph node status (positive vs negative). AUC = area under the receiver operating characteristic curve.

**Figure 5:**
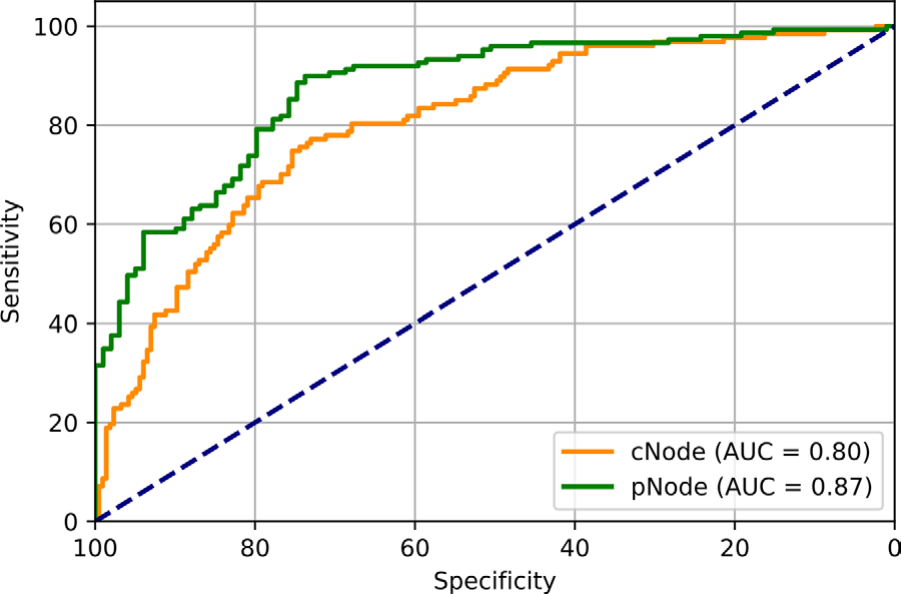
Diagnostic performance of two four-dimensional hybrid models using tumor plus axillary pixels versus tumor pixels alone in predicting pathologic node status (positive vs negative). *P* value was determined by DeLong test. AUC = area under the receiver operating characteristic curve, cNode = clinical node, pNode = pathologic node.

For the prediction of pN0 versus pN+ status, the 4D hybrid model yielded higher performance than the 3D model along the primary AUC performance measure with its AUC of 0.87 (95% CI: 0.83, 0.91) (*P* < .001) (Table 4). Additionally, the model produced a high sensitivity of 89% (95% CI: 79%, 93%), specificity of 76% (95% CI: 68%, 88%), and a low false-negative rate of 11% (7%, 21%) (Table 4). Table 5 summarizes the performance of the hybrid 4D model on the test data set at varying specificity cutoff points. At 71% specificity, the model yielded a sensitivity of 91% and a false-negative rate of 9%. At 51% specificity, sensitivity was 95% and the false-negative rate was 5%. Training and validation performances for the 4D hybrid model are shown in Table S4. The 4D hybrid model using tumor pixels alone had an AUC of 0.85, with sensitivity, specificity, and false-negative rates of 90% (95% CI: 85.5%, 94.7%), 64% (95% CI: 56.2%, 73%), and 9% (95% CI: 5.3%, 14.5%), respectively. Therefore, the use of axillary pixels improved model performance, though not significantly (*P* = .12) (Fig 4). Comparing the top-performing models that predict cN status to the model that predicts pN status, we find that for every specificity, the model predicting pN status attains higher sensitivity (Fig 5).

**Table 5:**
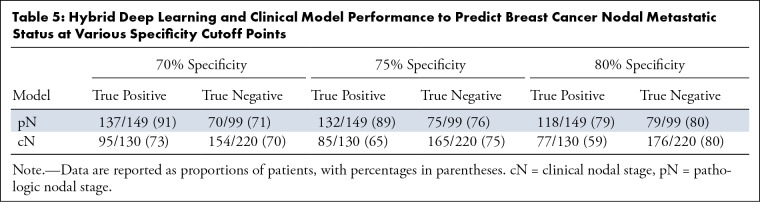
Hybrid Deep Learning and Clinical Model Performance to Predict Breast Cancer Nodal Metastatic Status at Various Specificity Cutoff Points

Of 17 patients with pN+ disease missed by the 4D hybrid model, 12 instances of pN+ disease (70.5%) were also missed by the radiologist, being identified as a single metastatic node at SLNB in patients with T1 ER-positive, HER2-negative cancers. The remaining five (30.5%) were identified with axillary imaging in patients with ER-positive (*n* = 3) or negative (*n* = 2) disease (cT1 = three and cT2 = two patients).

### Generalizability of the Model: Interinstitutional Comparison

To test the generalizability of our model, the 4D hybrid model for pN prediction was also trained from scratch using data from one institution at a time: a university hospital versus a safety-net hospital. The AUC, sensitivity, and specificity values of the safety-net hospital–trained model tested on the university hospital were 0.84 (95% CI: 0.75, 0.91), 73% (95% CI: 58%, 86%), and 91% (95% CI: 78%, 98%); for the university hospital–trained model tested on the safety-net hospital, the values were 0.81 (95% CI: 0.73, 0.87), 0.83% (95% CI: 0.6%, 0.97%), and 0.7% (95% CI: 0.49%, 0.9%), respectively.

### Saliency Mapping

**Figure 6:**
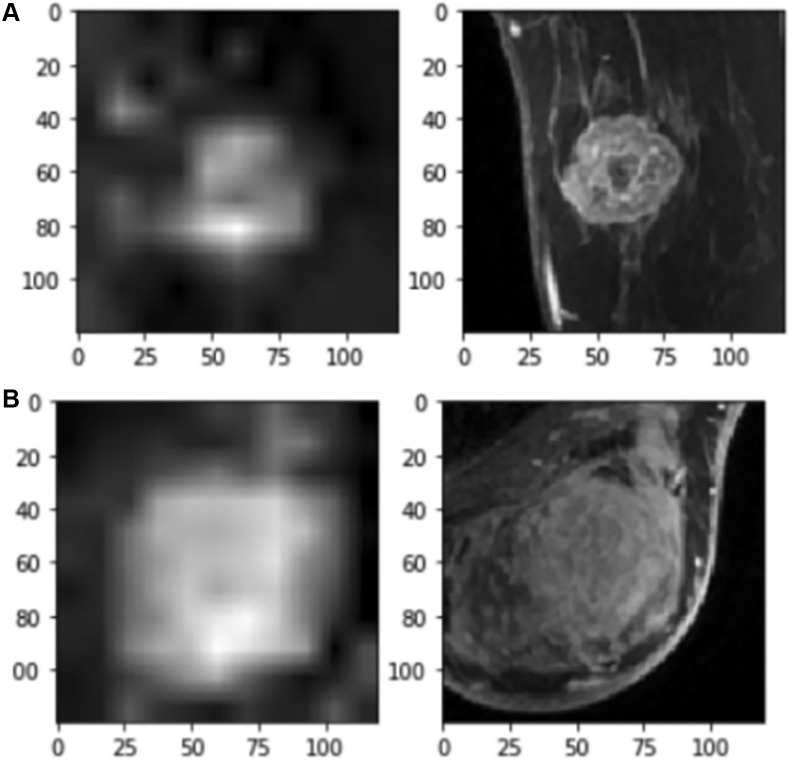
Saliency mapping. Important voxels are revealed through saliency mapping. Saliency mapping with gradient-weighted class activation mapping (left column) demonstrates that the primary active tumor voxels at MRI (right column) are those most valuable for prediction of both **(A)** nonmetastatic cancer and **(B)** axillary metastatic cancer.

Our saliency map shows that the model learns important features from the primary tumor and peritumoral voxels to predict absence (Fig 6A) or high probability of metastasis (Fig 6B). More distal voxels are less important. As the algorithm did not explicitly provide the nonlinear tumor boundary, this saliency map result suggests the model learned to approximate the tumor boundary on its own.

## Discussion

We developed a noninvasive imaging-based CNN model that helps determine axillary lymph node status in patients with breast cancer. Our MRI-based hybrid model showed AUC, sensitivity, and specificity values of 0.87, 89%, and 76%, respectively, which represents an improvement over the 77.6% radiologist sensitivity in our data set. One of the benefits of our model is its scalability. At a slightly lower specificity of 71%, our model yielded 91% sensitivity and a 9% false-negative rate, which align with the 90% sensitivity and less than 10% false-negative rate benchmarks used to define success for most SLNB studies, while maintaining higher diagnostic performances than axillary US, morphologic evaluation of DCE MRI, or other prognostics nomograms (34–36). The model using tumor pixels alone had comparable performance with our model (using both breast and axillary pixels), with a sensitivity of 90%, specificity of 64%, and false-negative rate of 9%. Hence, integration of our model into clinical practice can help triage a significant proportion of patients with low probability of metastasis with high sensitivity, mitigating additional diagnostic or invasive procedures and associated risk. Our model would have helped avoid more than half (51%) of benign sentinel node biopsies while correctly detecting 95% of patients with axillary metastasis. This is an important benefit, as some patients undergoing SLNB develop permanent complications, including lymphedema, seroma, wound infection, and pain, despite having a low probability of a positive result, which may substantially impact their management (37).

The diagnostic performance of our model represents an improvement over that of Arefan et al (26), which yielded sensitivity, specificity, and AUC of 82%, 78%, and 0.82, respectively. In a similar study that used DCE MR images from both tumor and axillary nodes (24,26) to develop a predictive clinical-radiomics algorithm for axillary metastasis detection, Yu et al (24) achieved an AUC of 0.9. However, the primary cancer pixel data alone yielded much lower diagnostic performance (AUC: 0.6). Segmentation and delineation of axillary nodes, as performed in their study, is a time-consuming process that is neither practical nor applicable in daily clinical practice. In contrast, our model yields a similar diagnostic performance but requires only a rough bounding box around the tumor without the need for precise tumor delineation. Adding the axillary image zone to our 4D hybrid model using tumor pixels alone improved its performance from 0.85 to 0.87, though not significantly (*P* = .12), with improved specificity (76% vs 64%) and no significant change in sensitivity (89% vs 90%), indicating that our model learns primarily from tumor images. This is an important advantage because the entire axilla may not consistently be included within the imaging field at standard breast MRI. Furthermore, this increases the day-to-day clinical applicability of our model by eliminating the need to segment out the axillary images, a process that involves extensive manual delineation.

In 2020, we showed that a 4D CNN can achieve high diagnostic accuracy to predict clinical lymph node stage (cN) across primary breast cancers (38). That preliminary study was performed using data obtained in a single hospital, with one MRI scanner and one MRI protocol. In our current work, we incorporate data from multiple hospitals, scanners, and protocols. The increased sample diversity likely improves our model’s generalizability to unseen data in the clinic. Additionally, in our current work, we introduced multiple architectural improvements to our algorithm, including a vision transformer–inspired patch-based approach (39) and improved hyperparameter optimization with HEBO (40) rather than traditional Bayesian optimization and hyperband (41). This improved model performance on cN prognostics and enabled us to develop the high-performing pN prognostic method that is unique to our current work.

In a recent study, Wang et al (42) used diffusion-weighted imaging and T1- and T2-weighted images to develop a CNN model, which yielded exceptional diagnostic performance (AUC: 0.996). In their study, the authors included “precursor lesions” that do not have metastatic potential, and unlike our study, they did not eliminate patients with cN0 disease who underwent neoadjuvant therapy. This is important, as existing axillary metastasis in these patients may have been eradicated over the course of treatment, making reference standard pathology unreliable. Additionally, their results are based on a single fold and thus could be inflated due to a fortuitous partition. While race information was not provided, with data originating from a single MRI unit and institution, lack of diversity may be pervasive. Additionally, no information on data preprocessing was provided.

Compared with previous models, our model has several advantages: *(a)* We partition the data into three parts (training, validation, and testing) through nested cross-validation, and the results we report pertain to the held-out test partition, which was not used during training and model selection. Reporting validation performance tends to overestimate the performance a model will achieve, while our more rigorous approach of reporting test performance provides a more realistic estimate of real-world clinical performance. *(b)* Our results are reported using nested fivefold cross-validation. As such, all data enter the test partition once. Compared with the single hold-out approaches used in the literature, our approach is not subject to a favorable or unfavorable partitioning result. *(c)* The deep learning approach we employ is unbiased by manual feature selection and instead directly learns optimal features for the imaging data, which is likely why our model does better on the tumor-only pixels than the model developed by Yu et al (24). Our model also compares well to published results using US, where original research (25) has revealed that a model trained on US images can yield AUC, sensitivity, and specificity values of 0.89, 85%, and 73%, respectively. However, the footprint of a US breast transducer is only approximately 4 cm, making the training of such models based on US images problematic for large tumors. Our results suggest that a machine learning model using DCE MRI to inform axilla involvement may be a safe and efficient tool for evaluating breast cancer, obviating further axillary imaging or diagnostic/surgical intervention.

We observed that using a sequence of 3D difference images rather than a single 3D difference image improved the AUC of the cN prediction (cN0 vs cN+) from 0.67 to 0.70 and improved pN prediction by a similar amount (from 0.69 to 0.71). Hence, the majority of the information learned by the model is contained in the spatial pattern of intensity differences (time 2 − time 0) and further contained in the combination of the clinical and imaging data, which bolsters performance to 0.79 and 0.87 AUC for cN and pN predictions, respectively.

Our data set had a well-balanced representation of patient race, with 36.3% of our patients being non-Hispanic White, 35% Hispanic, and 26% Black, which is an important consideration while developing CNN models applicable to patients of varying races and ethnicities. While predicting on more ethnically and potentially genetically heterogeneous data sets is more challenging, it is well representative of real-world performance in a heterogeneous population.

Our study had some limitations. The main limitation toward clinical adoption of our model is the current requirement for a manual coarse bounding box around the tumor area by the radiologist. Development and implementation of a fully automatic machine learning–based bounding box tool can allow time-efficient analysis of larger data sets. Another potential limitation was that currently, our data source consists of two affiliated hospitals using two different vendors. While overall model performance on the university hospital versus safety-net hospital were similar, the model trained on the safety-net hospital and tested on the university hospital had higher AUC and specificity compared with the model trained on the university hospital and tested on safety-net hospital data (AUC 0.84 vs 0.81, sensitivity 73% vs 83%, specificity 91% vs 71%). This illustrates the importance of incorporating imaging data from more institutions and different types of MRI units to further increase the variability of our data and enhance the generalizability of our results. In addition, the utilized scans originated from two 1.5-T MRI units, which are used in routine breast MRI in our institution; this may limit the applicability of our data to patients scanned at 3 T. As expected, the prevalence of pN2 and pN3 tumors was much lower than that of N0 and N1, as we excluded patients who underwent neoadjuvant chemotherapy. Hence, we used a binary classification, N0 versus N+ (includes N1, N2, and N3), to prevent any bias and skewing. Additionally, while we propose our best-performing model for further analysis, an independent prospective external validation would be welcome. Last, routine use of breast MRI in patients with breast cancer is still under debate. Patients with dense breasts, hormone receptor–negative disease, or unfavorable tumor subtypes eligible for neoadjuvant therapy may benefit more from the proposed model due to the higher significance and potential impact of axillary metastasis on their care.

In this study, we developed and validated a hybrid clinical and 4D MRI-based model that provides individualized prediction of axillary metastasis without the need for dedicated axillary imaging or invasive procedures. Our model is a safe and time-efficient tool, achieving noteworthy results compared with the existing methods. In the future, we look forward to additional optimization of the image analysis process and the inclusion of more variable data to further enhance the utility of the proposed models.
